# Differential diagnosis of localized pneumonic-type lung adenocarcinoma and pulmonary inflammatory lesion

**DOI:** 10.1186/s13244-022-01200-z

**Published:** 2022-03-22

**Authors:** Qi Li, Xiao Fan, Ji-wen Huo, Tian-you Luo, Xing-tao Huang, Jun-wei Gong

**Affiliations:** 1 Department of Radiology, The First Affiliated Hospital of Chongqing Medical University, No. 1 Youyi Road, Yuzhong District, Chongqing, 400016 China; 2 Department of Radiology, Children’s Hospital of Chongqing Medical University, No. 136 Zhongshan Road Two, Yuzhong District, Chongqing, 400014 China; 3 Department of Radiology, The Fifth People’s Hospital of Chongqing, No. 24 Renji Road, Nan’an District, Chongqing, 400062 China

**Keywords:** Pneumonic-type lung cancer, Adenocarcinoma, Inflammation, Computed tomography

## Abstract

**Background:**

In clinical practice, a number of delayed diagnoses of localized pneumonic-type lung adenocarcinoma (L-PLADC) mimicking pneumonia have been identified due to the lack of knowledge regarding the radiological findings associated with this condition. Here, we defined L-PLADC as a special type of lung adenocarcinoma that presents as a focal consolidation involving < 50% of the area of a lobe and aimed to investigate the differential clinical and imaging features between L-PLADC and localized pulmonary inflammatory lesion (L-PIL).

**Results:**

The data of 120 patients with L-PLADC and 125 patients with L-PIL who underwent contrast-enhanced chest computed tomography (CT) scan were retrospectively analyzed. For clinical characteristics, older age, women, nonsmokers, and no symptom were more common in L-PLADC (all *p* < 0.001). With regard to CT features, air bronchogram, irregular air bronchogram, ground-glass opacity (GGO) component, and pleural retraction were more frequently observed in L-PLADC, while necrosis, satellite lesions, halo sign, bronchial wall thickening, interlobular septa thickening, pleural attachment, and pleural thickening were more commonly seen in L-PIL (all *p* < 0.001). Multivariate analysis showed age ≥ 58 years, female sex, GGO component, irregular air bronchogram, pleural retraction, and the absence of necrosis and pleural attachment were the most effective variations associated with L-PLADC with an AUC of 0.979. Furthermore, an external validation cohort containing 62 patients obtained an AUC of 0.929.

**Conclusions:**

L-PLADC and L-PIL have different clinical and imaging characteristics. An adequate understanding of these differential features can contribute to the early diagnosis of L-PLADC and the subsequent therapeutic strategy.

## Key points

- The model incorporating clinical and CT features can differentiate L-PLADC from L-PIL with a relatively high accuracy.
- Focal consolidation with irregular air bronchogram, GGO component, pleural retraction, and the absence of necrosis and pleural attachment should raise suspicion of cancer.
- Familiarity with these differential features may contribute to an early diagnosis of L-PLADC.

## Introduction

Lung cancer is one of the most frequent malignancies worldwide and considered a major cause of cancer death, with the most common histological type being lung adenocarcinoma (LADC) [1]. This type has many faces on computed tomography (CT), including a solitary nodule or mass, a thin-walled cystic lesion, focal or diffuse parenchymal consolidation, or multifocal lesions [2–11]. When LADC manifests as parenchymal consolidation, it is often difficult to distinguish from pneumonia, leading to delayed diagnosis. Hence, this type of LADC is also known as pneumonic-type lung adenocarcinoma (PLADC), which refers to primary lung adenocarcinoma with a radiological pneumonic presentation and usually presents consolidative opacities [10, 11].

PLADC is a heterogeneous disease in which different patterns might be identified based on clinical, imaging, and histopathological manifestations. Our previous study classified PLADC into localized pneumonic-type lung adenocarcinoma (L-PLADC) defined as PLADC with a focal consolidation involving < 50% of the area of a lobe and diffuse PLADC characterized as diffuse consolidation involving ≥ 50% of the area of a lobe or lobes based on imaging, and found that PLADC with different ranges exhibited different clinical, imaging, and pathological characteristics [12]. Recently, many investigators have studied the clinical and radiological features of PLADC [4–11]. However, most patients included in these studies had diffuse PLADC [5–8, 10], and only minimal research has focused on patients with L-PLADC [9, 11]. In addition, a large number of delayed diagnoses of L-PLADC mimicking pneumonia were identified; this is largely attributable to a poor understanding of the radiological findings associated with L-PLADC.

Therefore, the present study aimed to evaluate the differential clinical and imaging features between L-PLADC and localized pulmonary inflammatory lesion (L-PIL).

## Materials and methods

### Patients

The study protocol was approved by the ethics committee of the First Affiliated Hospital of Chongqing Medical University, and the need for informed consent was waived because of the retrospective nature of this research. L-PLADC was identified using the following diagnostic criteria via CT: (1) pneumonia-like consolidation, characterized by an increased intensity of lung parenchyma with the obscuration of the underlying vessels and with a shape that could not be classified as round, oval, or lobulated (e.g., triangular, rectangular, or trapezoidal); (2) isolated and localized lesion, the largest slice of which involved less than half the area of a lobe on axial images.

From June 2015 to August 2021, we collected the data of 120 patients with L-PLADC who met the following inclusion criteria: (1) pathological confirmation of LADC; (2) contrast-enhanced chest CT scan; (3) and absence of multiple primary lung cancer. In addition, 125 patients with localized pulmonary inflammatory lesion (L-PIL) admitted to our hospital were also enrolled, including 48 with inflammatory pseudotumor, 40 with nonspecific inflammation, 20 with granulomatous inflammation, and 17 with focal-organizing pneumonia. These patients fulfilled the following inclusion criteria: (1) pathological confirmation of inflammatory lesion; (2) contrast-enhanced chest CT scan; (3) CT results showing focal consolidation involving less than half the lobe area; and (4) consolidation without obvious absorption on follow-up CT after 2–4 weeks of anti-inflammatory treatment. Patients with any anti-tumor or anti-inflammatory therapy prior to the initial chest CT scan were excluded. Furthermore, 62 patients admitted to another center from January 2018 to August 2021 who fulfilled the aforementioned inclusion and exclusion criteria were included as external validation cohort.

### CT protocols

Chest CT examinations were performed using Discovery CT750HD (GE Healthcare) or Somatom Definition Flash (Siemens Healthcare) scanner. Unenhanced CT scanning was first performed from the thoracic inlet to the lung base in the supine position. The following imaging parameters were used: tube voltage, 120 kVp; tube current, 100–250 mA; and scanning slice thickness/interval, 5 mm/5 mm. The patients were then injected with a nonionic iodinated contrast medium (iohexol 300 mg iodine/mL; Omnipaque, GE Healthcare) at a dosage of 1.5 mL/kg of body weight (total volume: 80–110 mL) using a dual high-pressure injector via the antecubital vein at a flow rate of 3.0 mL/s. This was followed by a 50-mL injection of saline solution at the same flow rate. Acquisition times in the arterial and delayed phases were triggered at 30 and 120 s, respectively, after the start of contrast medium injection. Subsequently, all unenhanced CT images were reconstructed with a slice thickness and slice interval of 0.625 mm or 1 mm and 0.625 mm or 1 mm, respectively, for imaging analysis.

### CT image analysis

Two experienced radiologists with > 10 years of experience in chest imaging interpreted the CT images together on a picture archiving and communication system (PACS) workstation (Vue PACS, Carestream). Any disagreements were resolved by discussion until a consensus was reached. The CT features of the lesions were carefully analyzed as follows:

1. Location of lesions (left upper and lower lobes as well as right upper, middle, and lower lobes).
2. Size (longest diameter of lesions in the lung window setting).
3. Margin (well-defined [clear border definition] and ill-defined [partially or completely blurred border definition]).
4. Internal characteristics (air bronchogram [branched or tubular air structure within lesions], irregular air bronchogram [air-filled bronchus manifesting as dilatation, rigidity, or narrowing], air-space [round or oval air attenuation within lesions], ground-glass opacity (GGO) [increased attenuation without the obscuration of the underlying lung vessels] component, necrosis [focal area of low attenuation without enhancement], and calcification). A GGO component refers to GGO mixed with consolidation or well-defined GGO around consolidation.
5. External characteristics (satellite lesions [lesions around consolidation with the distance from consolidation ≤ 3 cm], halo sign [ill-defined GGO around consolidation], thickened bronchial wall proximal to lesion, adjacent interlobular septa thickening, pleural retraction [linear structures connected between the lesion and pleura], pleural attachment [lesion attaches to the pleura with the margin obscured by the pleura], and adjacent pleural thickening).
6. Associated findings (lymphadenopathy [hilar or mediastinal lymph nodes with short-axis diameter > 1 cm] and pleural effusion).

For patients with sequential CT scans, image analyses were based on the earliest CT data that conformed to the diagnostic criteria of L-PLADC before treatment.

### Statistical analyses

Statistical analyses were performed using IBM SPSS Statistics for Windows (version 19.0; IBM Corp., Armonk, NY, USA). Single-sample Kolmogorov–Smirnov analysis was used to test the variance in the homogeneity of age, which was presented as medians ± interquartile range due to non-normal distribution. The Mann–Whitney U test was used to compare age between the groups and the Youden index (YI) (YI = sensitivity + specificity − 1) of age was calculated by Receiver operating characteristic (ROC) analysis and the maximum value was used to establish the optimal threshold value to distinguish L-PLADC from L-PIL. Categorical variables were expressed as numbers and percentages and the chi-squared test was used to compare age, sex, smoking history, respiratory symptoms, laboratory results, and CT features between the groups. Moreover, Multiple logistic regression analysis was performed using clinical and CT characteristics that differed significantly between groups to identify independent factors that can be used to diagnose L-PLADC. The final regression model was selected using the forward condition method, and the area under the curve (AUC), sensitivity, specificity, and accuracy were used to evaluate its diagnostic performance. A two-tailed *p* value of < 0.05 was considered statistically significant.

## Results

### Study population

**Fig. 1:**
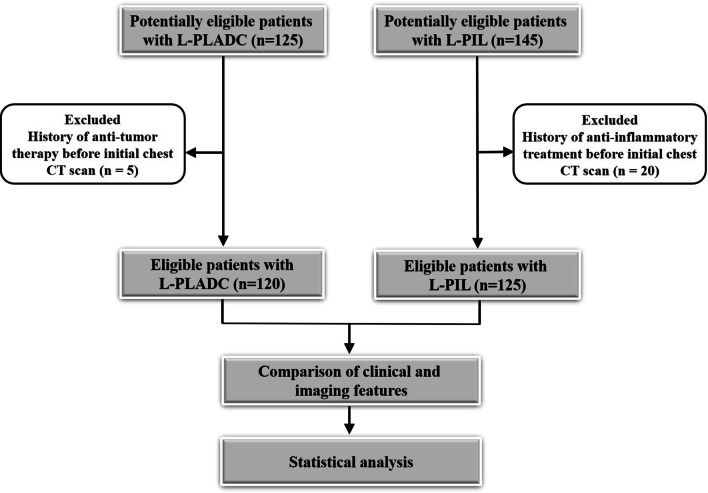
The flow diagram for this study

A total of 125 patients with L-PLADC and 145 patients with L-PIL were initially included. Among these, 25 patients were excluded owing to a history of anti-tumor or anti-inflammatory therapy before the initial chest CT scan. Finally, 120 patients with L-PLADC and 125 patients with L-PIL were included for analysis. The flow diagram of this study is shown in Fig. 1. Among the 120 patients with L-PLADC, 104 (86.67%) were diagnosed by surgical resection, 10 (8.33%) by bronchoscopy biopsy, 4 (3.33%) by transthoracic needle biopsy, and 2 (1.67%) by the cytological examination of sputum or pleural effusion. For the 104 patients undergoing surgical resection, all were confirmed with invasive LADC, including 79 (75.96%) with the acinar-predominant subtype, 10 (9.62%) with the papillary-predominant subtype, 5 (4.81%) with the lepidic-predominant subtype, 4 (3.85%) with the solid-predominant pattern, 4 (3.85%) with the histological subtype of invasive mucinous adenocarcinoma, and 2 (1.92%) with the micropapillary-predominant pattern. Ninety-five patients (79.17%) were in stages I–II and 25 (20.83%) were in stages III–IV at the time of diagnosis. All 125 patients with L-PIL were diagnosed by surgical resection. Similarly, 28 patients with L-PLADC and 34 patients with L-PIL in external validation cohort were confirmed by surgery.

### Comparison of clinical characteristics between L-PLADC and L-PIL

The clinical data of patients with L-PLADC and L-PIL are summarized in Table 1. For patients with L-PLADC, the ages ranged from 35 to 80 years, with a mean (± IQR) of 64 ± 13 years, while for patients with L-PIL, the ages ranged from 22 to 86 years, with a mean (± IQR) of 55 ± 16 years. The patients with L-PLADC were older than those with L-PIL (*p* < 0.001). The optimal cut-off value of age for differentiating L-PLADC from L-PIL was 57.5 years old, with an AUC, sensitivity, and specificity of 0.652, 70.8%, and 53.6%, respectively. Age ≥ 58 years, female sex, nonsmoking, and no respiratory symptom were more frequently observed in patients with L-PLADC than in those with L-PIL (all *p* < 0.001). However, no significant difference was observed in the white blood cell count between the groups (*p* > 0.05).

**Table 1:** Comparison of clinical characteristics between localized pneumonic-type lung adenocarcinoma and pulmonary inflammatory lesion

Characteristics	Patients with L-PLADC (*n* = 120)	Patients with L-PIL (*n* = 125)	*p* value
Age			< 0.001^a^
< 58 years	35 (29.17%)	67 (53.60%)	
≥ 58 years	85 (70.83%)	58 (46.40%)	
Gender			< 0.001^a^
Male	36 (30.00%)	89 (71.20%)	
Female	84 (70.00%)	36 (28.80%)	
Smoking history			< 0.001^a^
Nonsmokers	91 (75.83%)	55 (44.00%)	
Smokers	29 (24.17%)	70 (56.00%)	
Respiratory symptoms			< 0.001^a^
With symptoms	63 (52.50%)	101 (80.80%)	
Fever	6	15	
Cough	48	82	
Sputum	36	68	
Blood in sputum	12	28	
Hemoptysis	8	24	
Chest pain	14	25	
Without symptoms	57 (47.50%)	24 (19.20%)	
Elevation of white blood cell count	13 (10.83%)	23 (18.40%)	0.094^a^

*L-PLADC*—localized pneumonic-type lung adenocarcinoma, *L-PIL*—localized pulmonary inflammatory lesion

^a^Chi-squared test

### Comparison of CT features between L-PLADC and L-PIL

**Fig. 2:**
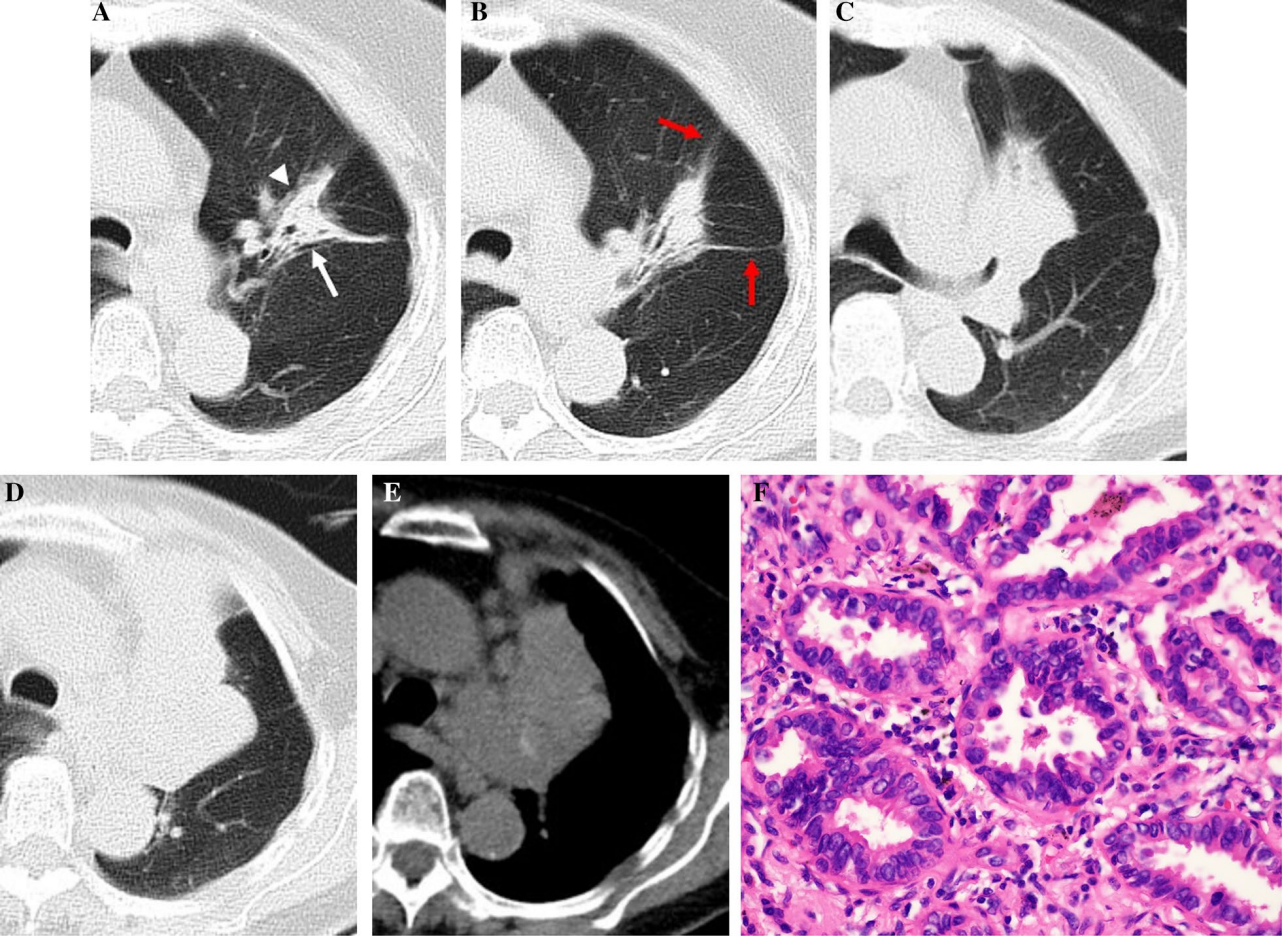
L-PLADC in an 83-year-old woman without symptoms. **A** and **B** Axial CT images of lung window indicate a localized consolidation with irregular air bronchogram (white arrow), GGO component (white arrowhead), and pleural retraction (red arrow) in the left upper lobe. This patient was misdiagnosed as inflammatory lesion and did not receive further examination. **C**–**E** Thirty-eight months later, follow-up CT scan shows a hilar mass in the left upper lobe with mediastinal lymphadenopathy. **F** Photomicrograph (hematoxylin and eosin staining, × 400) confirmed LADC with an acinar-predominant pattern

**Fig. 3:**
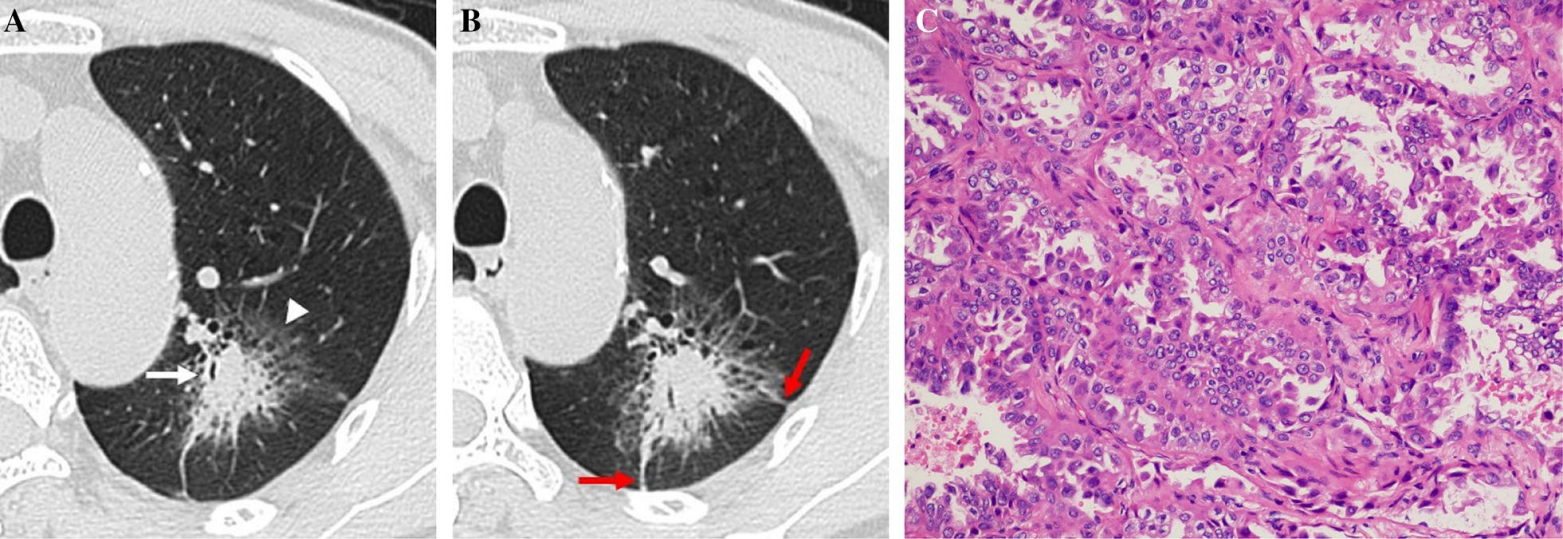
L-PLADC in a 68-year-old man without symptoms. **A** and **B** Axial CT images of lung window indicate a localized consolidation with irregular air bronchogram (white arrow), GGO component (white arrowhead), and pleural retraction (red arrow) in the left upper lobe. **C** Photomicrograph (hematoxylin and eosin staining, × 200) confirmed LADC with an acinar-predominant pattern

**Fig. 4:**
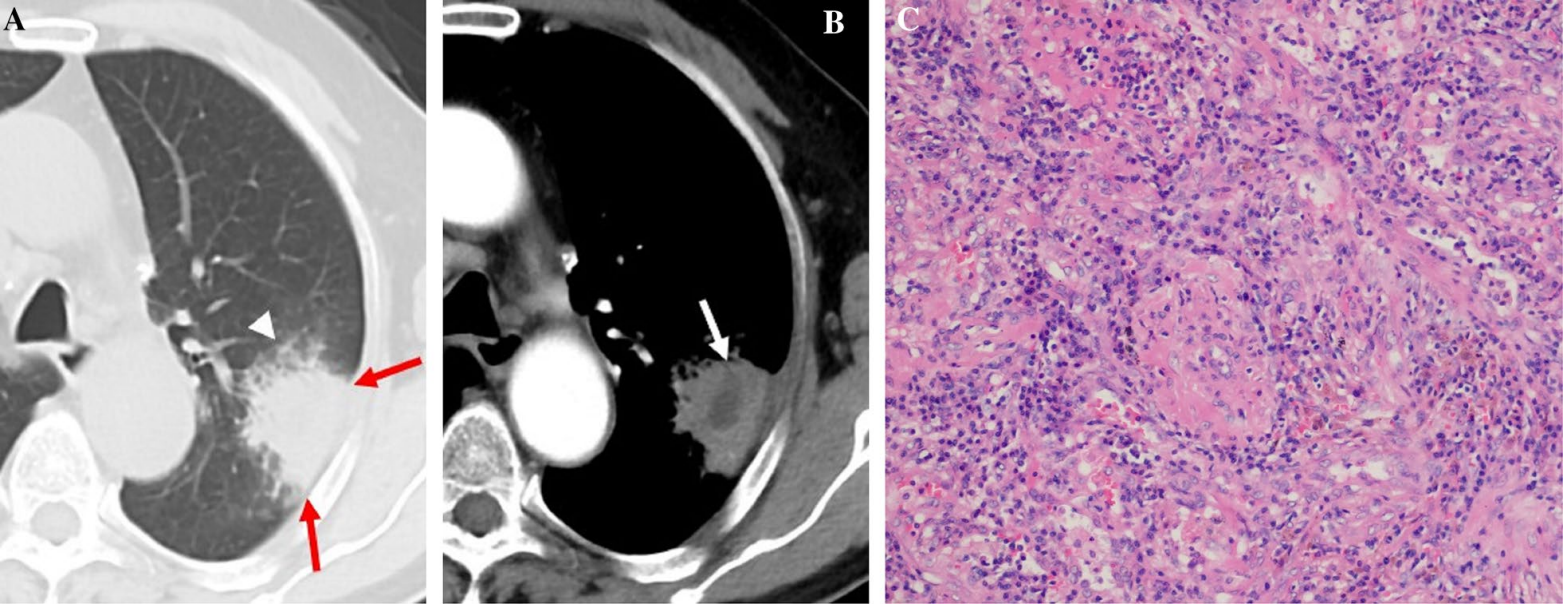
L-PIL in a 48-year-old man with left chest pain for 12 days. **A** Axial CT image of the lung window indicates localized consolidation with halo sign (white arrowhead) and pleural attachment (red arrow) in the left upper lobe. **B** Axial CT image of the mediastinal window in arterial phase indicates necrosis (white arrow) within the lesion. **C** Photomicrograph (hematoxylin and eosin staining, × 100) confirmed nonspecific inflammation

**Fig. 5:**
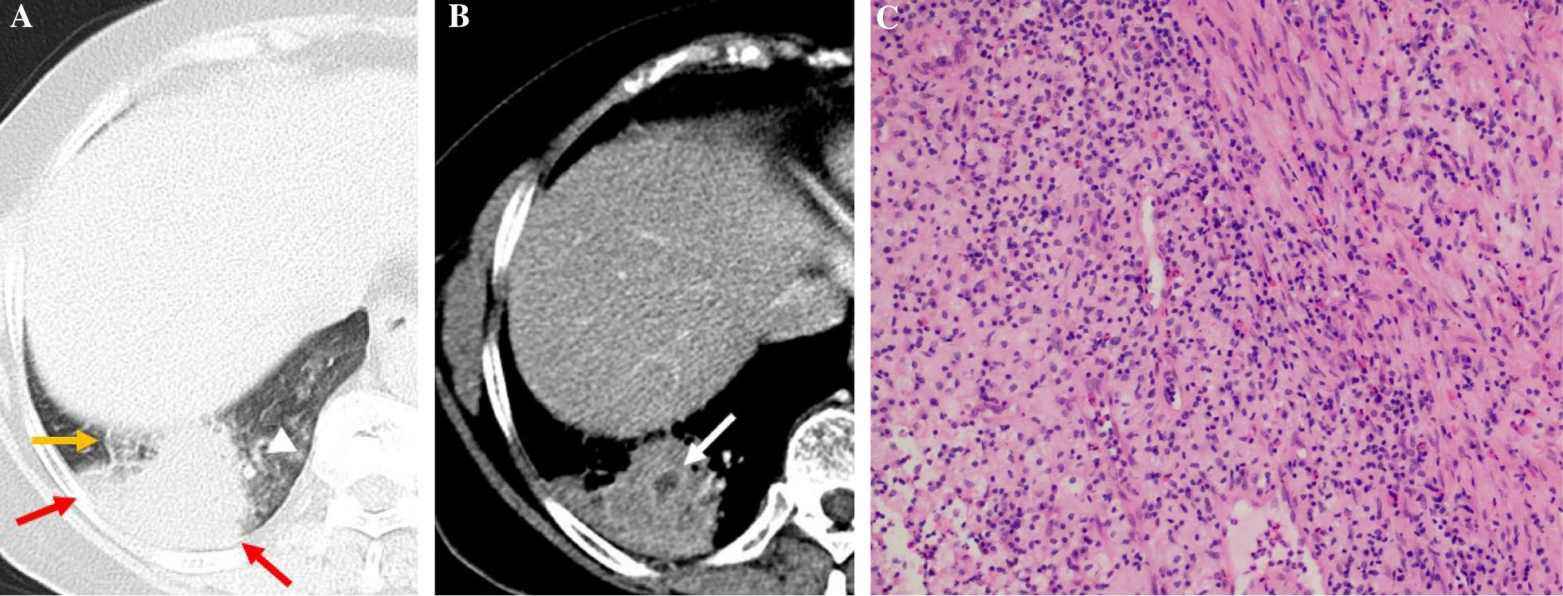
L-PIL in a 66-year-old woman with cough and hemoptysis for two weeks. **A** Axial CT image of the lung window indicates localized consolidation with halo sign (white arrowhead), adjacent interlobular septa thickening (yellow arrow), and pleural attachment (red arrow) in the right lower lobe. **B** Axial CT image of the mediastinal window in arterial phase indicates necrosis (white arrow) within the lesion. **C** Photomicrograph (hematoxylin and eosin staining, × 100) confirmed inflammatory pseudotumor

Table 2 summarizes the CT features of the two groups. The mean tumor size was 3.64 ± 1.39 cm (range, 2.27–9.35 cm), whereas that of the inflammatory lesions was 4.25 ± 1.77 cm (range, 2.42–8.86 cm). Air bronchogram, irregular air bronchogram, GGO component, and pleural retraction were more common in the L-PLADC group, whereas necrosis, satellite lesions, halo sign, bronchial wall thickening, interlobular septa thickening, pleural attachment, and adjacent pleural thickening were more frequently observed in the L-PIL group (all *p* < 0.001) (Figs. 2, 3, 4, 5). However, no significant differences were observed between the groups with respect to location, margin, air-space, calcification, lymphadenopathy, and pleural effusion (all *p* > 0.05).

**Table 2:** Comparison of CT features between localized pneumonic-type lung cancer and pulmonary inflammatory lesion

CT features	Patients with L-PLADC (*n* = 120)	Patients with L-PIL (*n* = 125)	*P* value
Location			0.414^a^
The upper lobe	79 (65.83%)	76 (60.80%)	
Right upper lobe	45	43	
Left upper lobe	34	33	
The middle and lower lobes	41 (34.17%)	49 (39.20%)	
Right middle lobe	11	12	
Right lower lobe	18	25	
Left lower lobe	12	12	
Margin			0.178^a^
Well-defined	81 (67.50%)	74 (59.20%)	
Ill-defined	39 (32.50%)	51 (40.80%)	
Internal characteristics			
Air bronchogram	102 (85.00%)	43 (34.40%)	< 0.001^a^
Irregular air bronchogram	82 (68.33%)	23 (18.40%)	< 0.001^a^
Air-space	37 (30.83%)	41 (32.80%)	0.741 ^a^
GGO component	75 (62.50%)	6 (4.80%)	< 0.001^a^
Necrosis	2 (1.67%)	65 (52.00%)	< 0.001^a^
Calcification	8 (6.67%)	9 (7.20%)	0.870 ^a^
External characteristics			
Satellite lesions	14 (11.67%)	48 (38.40%)	< 0.001^a^
Halo sign	23 (19.17%)	86 (68.80%)	< 0.001^a^
Adjacent bronchial wall thickening	3 (2.50%)	47 (37.60%)	< 0.001^a^
Adjacent interlobular septa thickening	5 (4.17%)	31 (24.80%)	< 0.001^a^
Pleural retraction	94 (78.33%)	50 (40.00%)	< 0.001^a^
Pleural attachment	49 (40.83%)	115 (92.00%)	< 0.001^a^
Adjacent pleural thickening	23 (19.17%)	61 (48.80%)	< 0.001^a^
Associated findings			
Lymphadenopathy	13 (10.83%)	21 (16.80%)	0.177^a^
Pleural effusion	6 (5.00%)	10 (8.00%)	0.342^a^

*CT*—computed tomography, *L-PLADC*—localized pneumonic-type lung adenocarcinoma, *L-PIL*—localized pulmonary inflammatory lesion, *GGO*—ground glass opacity

^a^Chi-squared test

### Multivariate analysis

For the model with clinical and CT characteristics that differed significantly between the groups, age ≥ 58 years (odds ratio [OR] 1.109; *p* < 0.001); female sex (OR 5.079; *p* = 0.004); GGO component (OR 35.086; *p* < 0.001); irregular air bronchogram (OR 18.746; *p* < 0.001); pleural retraction (OR 4.561; *p* = 0.009); the absence of necrosis (OR 0.007; *p* < 0.001); and the absence of pleural attachment (OR 0.157; *p* = 0.008) were the independent predictors of L-PLADC via multivariate logistic regression analysis. The AUC, sensitivity, specificity, and accuracy for the logistic regression function (*p* = 1/(1 + e ^(7.817 − 0.103 *age≥58 years −1.625* female sex − 3.558 * GGO component − 2.931 * irregular air bronchogram + 4.981* necrosis − 1.517 * pleural retraction + 1.851* pleural attachment)^)) were 0.979, 91.1%, 93.4%, and 92.2%, respectively. The external validation cohort obtained an AUC, sensitivity, specificity, and accuracy of 0.929, 91.3%, 82.1%, and 85.5%, respectively.

### Morphological evolution of L-PLADC during follow-up CT

**Fig. 6:**
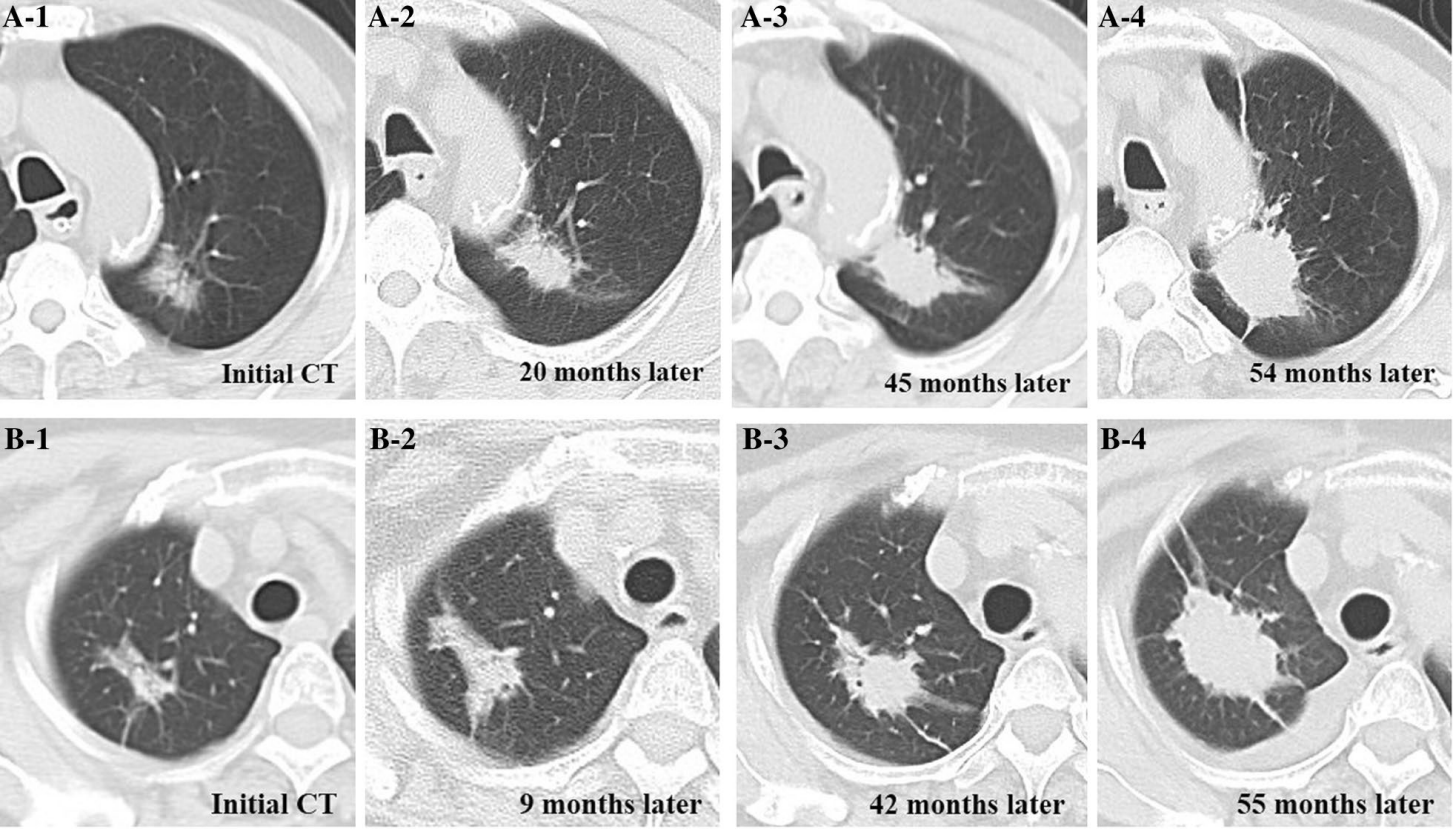
Morphological evolution of L-PLADC during follow-up CT in an 83-year-old man (**A**) and a 72-year-old woman (**B**), respectively. **A** and **B** The two patients were misdiagnosed as pulmonary inflammatory lesion on initial CT. Sequential CT images of the lung window indicate enlarged focal consolidation, increased attenuation, gradual disappearance of air bronchogram and GGO component; eventually, the focal consolidation became a solid mass during follow-up

Follow-up chest CT revealed that 14 patients with L-PLADC experienced disease progression. The average observation period was 22 months (range, 4–55 months), and the average follow-up interval was 11 months (range, 1–42 months). Among these patients, three had undergone four follow-up CT scans, seven had undergone three follow-up CT scans, and four had undergone two follow-up CT scans. The tumor diameter enlarged over time; attenuation increased; air bronchogram, air-space, and GGO component gradually disappeared; and the lesions ultimately became solid masses (Figs. 2, 6).

## Discussion

L-PLADC is characterized by a focal area of consolidation along with the presence of air bronchogram in most cases. These features mimic infection and may result in an erroneous diagnosis. In the present study, we compared the clinical and imaging characteristics between the L-PLADC and L-PIL groups and identified several distinctive features that can help assist in distinguishing these entities.

In terms of clinical characteristics, older age, female sex, nonsmoking, and no respiratory symptom were more common in patients with L-PLADC than in those with L-PIL. Some scholars [13] indicated that patients with lung cancers were older than those with inflammatory lesions, which is consistent with our findings. Many investigators have demonstrated that LADC is more likely to be associated with women and nonsmokers [14, 15]. As expected, we found that patients with L-PLADC were predominantly women and nonsmokers. Furthermore, nearly half of the patients with L-PLADC were asymptomatic; this result was similar to that observed in some studies [4, 13].

With regard to CT features, this study revealed that air bronchogram, irregular air bronchogram, GGO component, and pleural retraction favored the diagnosis of L-PLADC, whereas necrosis, satellite lesions, halo sign, bronchial wall thickening, interlobular septa thickening, pleural attachment, and pleural thickening were suggestive of L-PIL. Air bronchogram represents remnant bronchi within lesions and was much more prevalent in cancers, which was in agreement with the findings of a few studies [16, 17]. In general, tumor cells seldom obliterate the underlying pulmonary architecture at an early stage, including the bronchus within it, leading to air bronchogram on CT imaging. As the tumor grows, the bronchus stretches, narrows, or even disappears because of a desmoplastic reaction or tumor invasion, thereby resulting in irregular air bronchogram [18]. This coincides with the morphological evolution of L-PLADC exhibited herein, which indicated that air bronchogram gradually disappears with tumor growth. In the present study, we found a valuable sign, namely GGO component, characterized by GGO mixed with consolidation or well-defined GGO around consolidation. It was less mentioned in some studies with respect to the differentiation of benign and malignant lung lesions. Notably, it needs to be carefully distinguished from the halo sign, which refers to ill-defined GGO around consolidation and is frequently observed in inflammatory lesions [5, 6, 9, 13]. Several studies have indicated that invasive LADC can display five histological architectural patterns (lepidic, acinar, papillary, solid, and micropapillary). Invasive LADC typically manifests as a complicated mixture of these patterns, and different patterns may exhibit different densities on CT [18–21]. According to these studies, the lepidic, acinar, or papillary-predominant pattern may present with GGO on CT imaging, particularly the lepidic pattern in which tumor cells grow along the alveolar wall but do not completely fill the alveolar space [18–21]. In the present study, these growth patterns were commonly observed in L-PLADC, which may be a good explanation to the higher occurrence of GGO component in cancers. Pleural retraction was another common observation in L-PLADC, which may correlate with the infiltration of fibrous tissue produced by tumor cells along the subpleural stroma or tumor mesenchyme contraction. Further, our results showed that necrosis was detected more frequently in inflammatory lesions, which is similar to previous results [13]. Inflammatory necrosis is associated with inflammatory responses caused by microbial infection and is generally observed at an early stage, whereas tumoral necrosis results from chronic ischemic injury or hypoxia and is generally observed in large tumors and seldom occurs in L-PLADC involving localized areas [22]. Satellite lesions, halo sign, adjacent bronchial wall thickening, and interlobular septa thickening were commonly observed in inflammatory lesions and associated with the spread of inflammation along adjacent alveoli, bronchi, and interstitium. We found that pleural attachment and thickening were highly indicative of inflammatory lesions. The reason for this finding may be that the inflammatory exudation of alveoli easily disseminates to the subpleural area and leads to an inflammatory response in the pleura.

Multiparametric analysis indicated that age ≥ 58 years, female sex, GGO component, irregular air bronchogram, pleural retraction, and the absence of necrosis and pleural attachment were the most important independent prognostic factors for diagnosing L-PLADC. Familiarity with these differential features may contribute to the accurate diagnosis of L-PLADC and reduce the unnecessary surgical resection rate of L-PIL.

In the current study, 14 patients experienced disease progression at follow-up CT, and the tumors showed some morphological changes over time, which ranged from focal consolidation to irregular solid masses. Hence, we think that L-PLADC may be an early manifestation of LADC and focal consolidation with irregular air bronchogram, GGO component, and pleural retraction should raise suspicion of cancer. A follow-up CT after 8–12 weeks with or without empirical anti-infective therapy may be helpful in further determining whether the lesion is benign or malignant. If the lesion does not change, additional tests such as CT-guided percutaneous transthoracic biopsy are often required, while if the lesion progresses with the morphological changes observed herein, surgical resection should be considered.

This study has several limitations that should be considered. First, given that the majority of PLADC is found in adenocarcinomas, our analyses were limited to adenocarcinomas and did not include other histologic types. Second, the retrospective nature of this study might have resulted in selection bias. Third, because of the lack of long-term follow-up data, we did not perform the survival analysis of patients with L-PLADC. However, we expect that future studies will reveal the prognosis of patients with L-PLADC.

Taken together, our findings demonstrate that L-PLADC and L-PIL have different clinical and imaging characteristics. An adequate understanding of these differential features can contribute to the early diagnosis of L-PLADC and the subsequent therapeutic strategy.

## Data Availability

The datasets used and/or analyzed during the current study are available from the corresponding author on reasonable request.

## Abbreviations

GGO: Ground-glass opacity
LADC: Lung adenocarcinoma
L-PIL: Localized pulmonary inflammatory lesion
L-PLADC: Localized pneumonic-type lung adenocarcinoma
PACS: Picture archiving and communication system
PLADC: Pneumonic-type lung adenocarcinoma
